# Comparative mRNA and miRNA transcriptome analysis of a mouse model of IGFIR-driven lung cancer

**DOI:** 10.1371/journal.pone.0206948

**Published:** 2018-11-09

**Authors:** Robert A. Jones, Sarah E. Franks, Roger A. Moorehead

**Affiliations:** Department of Biomedical Sciences, Ontario Veterinary College, University of Guelph, Guelph, ON, Canada; University of South Alabama Mitchell Cancer Institute, UNITED STATES

## Abstract

Mouse models of cancer play an important role in elucidating the molecular mechanisms that contribute to tumorigenesis. The extent to which these models resemble one another and their human counterparts at the molecular level is critical in understanding tumorigenesis. In this study, we carried out a comparative gene expression analysis to generate a detailed molecular portrait of a transgenic mouse model of IGFIR-driven lung cancer. IGFIR-driven tumors displayed a strong resemblance with established mouse models of lung adenocarcinoma, particularly EGFR-driven models highlighted by elevated levels of the EGFR ligands *Ereg* and *Areg*. Cross-species analysis revealed a shared increase in human lung adenocarcinoma markers including *Nkx2*.*1* and *Napsa* as well as alterations in a subset of genes with oncogenic and tumor suppressive properties such as *Aurka*, *Ret*, *Klf4* and *Lats2*. Integrated miRNA and mRNA analysis in IGFIR-driven tumors identified interaction pairs with roles in ErbB signaling while cross-species analysis revealed coordinated expression of a subset of conserved miRNAs and their targets including *miR-21-5p* (*Reck*, *Timp3* and *Tgfbr3)*. Overall, these findings support the use of SPC-IGFIR mice as a model of human lung adenocarcinoma and provide a comprehensive knowledge base to dissect the molecular pathogenesis of tumor initiation and progression.

## Introduction

Lung cancer is the leading cause of cancer-related deaths worldwide [1]. Non-small cell lung cancer (NSCLC) represents the major form of the disease accounting for up to 85% of cases and can be broadly categorized into adenocarcinoma (ADC), squamous cell carcinoma (SCC) and large cell carcinoma (LLC) histological subtypes [2,3]. ADC is the most frequently diagnosed form of NSCLC and is characterized by glandular differentiation, expression of thyroid transcription factor 1 (TTF1 also known as NK2 homeobox 1 or NKX2-1) and frequent mutations in *KRAS* or *EGFR* [2,3]. SCC accounts for approximately 40% of NSCLC cases and can be distinguished from ADC by expression of p63 and basal keratins while LLC is less prevalent and diagnosed when features of ADC or SCC are absent [2,3].

Mouse models have been widely employed to explore the biology of human malignancies including lung cancer [4]. Models that permit transformation of normal cells in situ, for example those that rely on chemical or genetic methods of tumor initiation, have been of particular value. Given the link between smoking and lung cancer risk, chemically induced mouse models of lung cancer have been developed through administration of tobacco-related carcinogens such as urethane and 4-(methylnitrosamino)-1-(3-pyridyl)-1-butanone (NNK)[5]. Resulting tumors harbor features of NSCLC with adenoma (AD) or ADC histopathology and frequently harbor activating *Kras* mutations. More precise manipulation of candidate oncogenes and tumor suppressors have been achieved with genetically engineered mouse models (GEMMs). For instance, promoters that drive transgene expression to specific cell lineages including surfactant protein-C (SPC) which directs expression to type II alveolar cells (AT2) and Club cell secretory protein (CCSP) which targets non-ciliated Club cells allow for spatial control of transgene expression [6]. In addition, temporal control can be achieved with the Cre-loxP and tetracycline (tet) inducible systems [6]. Together, these advances have allowed for the development of more complex and accurate models of the disease.

The vast majority of GEMMs developed to date resemble lung ADC [4]. Given the frequent occurrence of *KRAS* mutations in human lung ADC, those based on mutant versions of *Kras* have been widely studied [4,7]. In the Kras^LA2^ model, spontaneous recombination of a latent mutant Kras allele (G12D) leads to the formation of lung tumors with complete penetrance and features of AD/ADC [8]. To allow for better control over tumor latency and multiplicity, conditional models of *Kras*-mutant lung cancer incorporating Cre- and/or tet-inducible alleles have also been developed [9–11]. In addition to their use in exploring the molecular mechanisms of tumor initiation and progression [9,10,12,13], *Kras*-mutant models have been valuable in the pre-clinical setting and have led to the identification of modifiers of treatment response [14,15] and therapeutic strategies to specifically target *Kras*- mutant cancers [16].

A series of tet-inducible models of mutant EGFR-driven lung ADC have also been generated [17–19]. Overexpression of an EGFR tyrosine kinase domain mutant (L858R) that is sensitive to the EGFR TKI erlotinib led to the rapid development of tumors that phenotypically resembled the human disease [17]. In contrast, mice in which an erlotinib resistant mutant (T790M) is overexpressed either alone or in combination with L858R resulted in tumor formation after long latency. These mutant strains have been particularly valuable for identifying novel therapeutic targets to overcome EGFR TKI resistance [18,20].

In addition to Kras and Egfr, the pro-tumorigenic role of other molecular alterations found in human lung ADC have been established in mice including the Ras signaling mediator cRaf-1 [21] and the pro-inflammatory transcriptional activator Stat3 [22]. Furthermore, recent advances in our understanding of lung cell lineage genes as well as genomic profiling have led to the development of models that resemble other subtypes of NSCLC. These models include adenosquamous carcinomas driven by combined loss of Pten and Smad4 [23], mucinous adenocarcinomas resulting from overexpression of Kras on a Nkx2.1 haploinsufficient background [24] and SCC following loss of the key tumor suppressors Lkb1 and Pten [25].

Understanding the degree to which mouse models of lung cancer resemble the human disease is crucial for the accurate translation of findings between species. While the Kras^LA2^ GEMM and urethane-induced models have been shown to share a common gene expression profile with human lung ADC [26,27] whether models carrying oncogenic drivers other than *Kras* reflect the molecular changes found in human lung ADC remains unclear. We have previously described a doxycycline inducible mouse model of lung cancer, SPC-IGFIR, in which the human type-I insulin-like growth factor receptor (IGFIR) is overexpressed AT2 cells via the SPC promoter [28]. These mice develop nodular lesions that resemble AD and ADC with a latency of approximately 9 months of age. Given that hyper-activation of IGFIR has been observed in NSCLC patient specimens [29] and disruption of IGFIR signaling with monoclonal antibodies or small molecules inhibits tumor growth *in vivo* [30,31], a better understanding of the molecular features of this model is warranted. Here, we present an mRNA and miRNA comparative analysis of the SPC-IGFIR model with other established mouse models and human lung cancers.

## Materials and methods

### Mice

The generation of SPC-IGF1R transgenic mice on an FVB background has been previously described[28]. To induce transgene expression, mice were fed chow supplemented with 2g/kg doxycycline (Harlan/Envigo) beginning at 21 days of age. For tissue collection, mice were sacrificed by CO_2_ asphyxiation. Mice were maintained following the Canadian Council for Animal Care guidelines and ethical approval was provided by the Animal Care Committee at the University of Guelph.

### RNA-Seq, miRNA-Seq and data analysis

Total RNA was extracted using the miRVana miRNA Isolation Kit (ThermoFisher, USA). Sequencing was carried out using an Illumina Hiseq 2000 sequencer at the McGill University and Genome Quebec Innovation Centre. Libraries were prepared using the firststrand TruSeq RNA and TruSeq small RNA protocols. For RNA-Seq, reads were trimmed and adapters removed using Trimmomatic aligned to the GRCm38 reference genome with STAR [32] and quantified with FeatureCounts [33]. For miRNA-Seq, reads were trimmed with Cutadapt [34], aligned with Bowtie1.01[35] and quantified with miRDeep (v2.0.0.7). To estimate mRNA expression of the human IGF1R transgene versus endogenous murine Igf1r levels, a hybrid reference genome (GRCm38 plus GRCh38) was generated for read alignment and a modified GTF was utilized for annotation.

#### Clustering and heatmap visualization

For hierarchical clustering and/or heatmap generation of RNA-Seq data, raw counts were first normalized for library size using the *estimateSizeFactors* function in the DESeq2 package[36]. The normalized counts were then log2 (plus 1 pseudo-count) transformed, gene-wise median centered and then clustered and visualized using the pheatmap package (v1.0.8) in R.

#### Differential gene expression analysis

For RNA-Seq datasets, differentially expressed genes were identified with DESeq2 (version 1.10.1)[36]. Raw count data for ADC, SCC and corresponding normal tissues from the LUAD and LUSC TCGA datasets was downloaded from GEO (GSE62944). Identification of differentially expressed genes from mouse model microarray datasets was carried out using the GEO2R tool for GEO datasets or limma [37] for datasets from EMBL-EBI Array Express (E-MEXP-1137) and the Lkb1/Pten model (kindly provided by Dr. Paul Hammerman). For miRNA analysis of the LSL-Kras^G12D^ model, p-values and isomiR (5p/3p-arm level) expression were not reported in the original publication[38]. Therefore, raw FASTQ files were downloaded from SRA (SRP017615), and processed and analyzed as above. Chromosome locations of miRNAs were extracted from the mmu.gff file provided by miRBase v21.

#### Gene set enrichment analysis (GSEA) and gene annotation enrichment

GSEA was carried out using the pre-ranked tool[39] as recommended for RNA-Seq data (https://software.broadinstitute.org/cancer/software/gsea/wiki/index.php/FAQ). Genes were ranked using the signed log10 transformed p-value. The following parameters were used: number of permutations = 1000, enrichment statistic = weighted, max gene set size = 500, min gene set size = 8. Gene-sets with an FDR value < 0.001 were considered significant. Gene-sets were obtained from http://baderlab.org/GeneSets and the MSigDB (v5.0). GO and KEGG enrichment analysis of gene lists were carried out using DAVID 6.8 [40]. Cytoscape and Enrichment Map[41] were used for visualization.

### qPCR

qPCR was carried out as previously described [42]. Primers were obtained from Bio-Rad (CA, USA) and relative quantification of gene expression was determined using the ΔΔCq method with *Hprt* and *Ywhaz* reference genes.

### Primary lung tumor cell isolation and magnetic activated cell sorting

Primary tumor tissue from SPC-IGF1R mice was dissected, minced and placed in 4 ml of 1mg/ml collagenase/dispase solution (Sigma-Aldrich) in RPMI-1640 (GIBCO) with shaking at 220rpm for 1.5hrs at 37°C. The digestion solution was replaced with fresh enzymes after 45 minutes. Digested tissue was then centrifuged for 5 min at 350xg, treated with 2U/ul of Dnase I (Sigma-Aldrich) and passed through a 40μM strainer. Contaminating red blood cells were then removed with RBC lysing buffer (Sigma-Aldrich) and cells were resuspended in HBSS supplemented with 2%FBS (GIBCO). Separation of epithelial and non-epithelial cells (CD45, CD31, Tert119 and BP-1) was carried out using the EasySep Mouse Epithelial Enrichment Kit (1:10 epithelial enrichment cocktail, 1:20 biotin selection cocktail and 1:10 magnetic particles) and EasySep Magnet (STEMCELL Technologies). To further enrich for tumor cells, a biotinylated anti-human IGF1R antibody (1:10, clone REA271, Miltenyi Biotech) was added to the epithelial enriched fraction for 30 min on ice followed by positive selection and separation as above.

### Statistics

Statistical analysis and visualization was performed using R-3.4.2 and Prism 5 (Graphpad, La Jolla, CA) unless otherwise stated. ANOVA followed by post-hoc Tukey test was used to compare means from multiple groups while unpaired or paired student’s t-test was used for the comparison of two means. Error is represented by standard error of the mean (s.e.m). Statistical significance is noted as p < 0.05.

## Results

### Gene expression profiling of murine IGFIR-driven lung tumors

**Fig 1:**
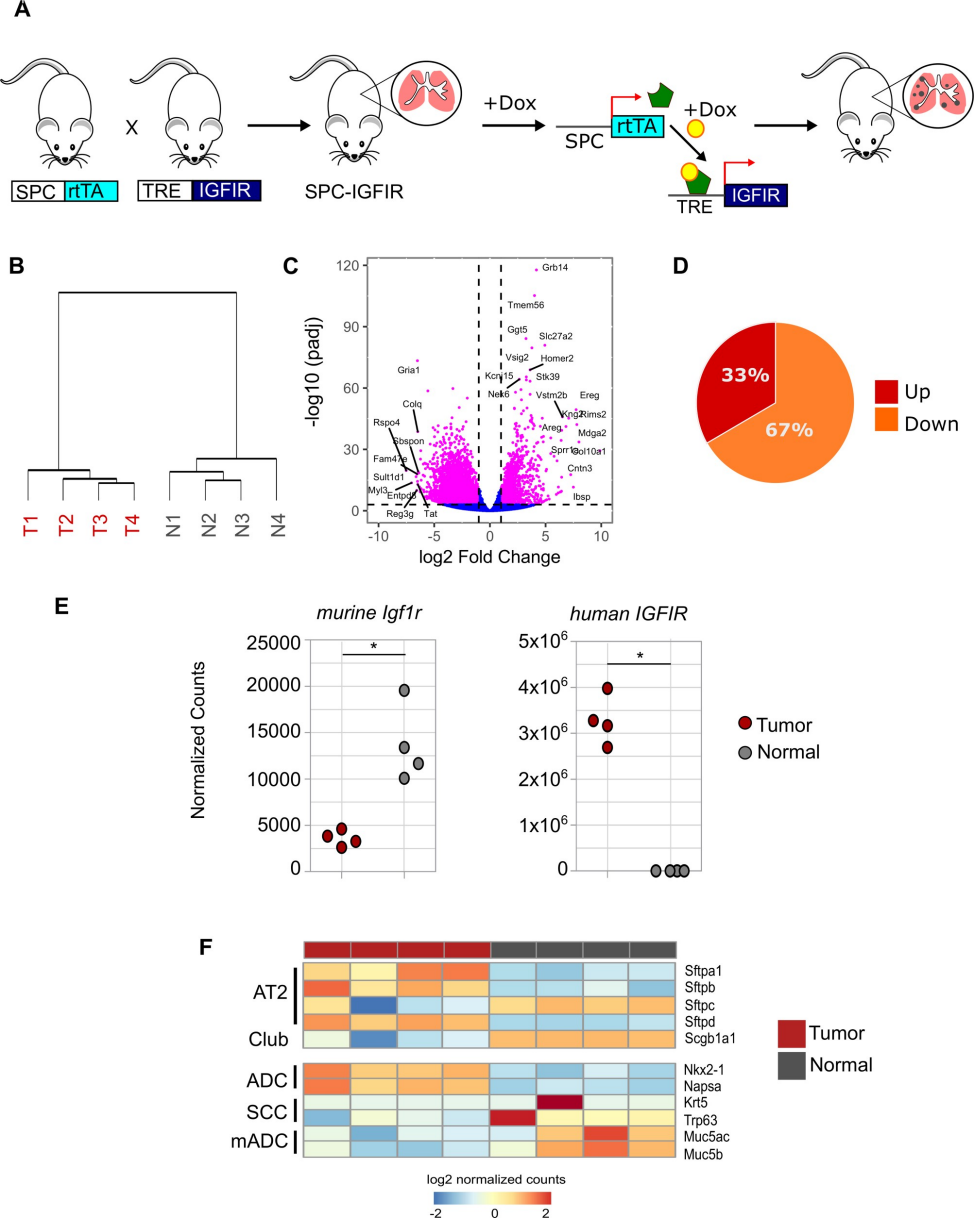
RNA-Seq analysis of SPC-IGFIR mice. **(A)** Schematic of doxycycline inducible expression of IGFIR in the mouse lung via the SPC promoter. **(B)** Unsupervised hierarchical clustering dendrogram of RNA-Seq data from tumor (T, red) and non-transgenic normal lung (N, grey) samples. **(C)** Volcano plot of log2 fold changes and differential expression p values between tumor and normal lung tissue. **(D)** Pie chart illustrating percentage of genes up and down-regulated in IGFIR-driven tumors. **(E)** Dot plots of endogenous murine *Igf1r* (padj = 5.71E-11) and human *IGFIR* transgene (padj = 5.65E-249) mRNA expression following mapping to a hybrid genome. **(F)** Heatmap showing differential expression of markers of AT2 and Club cells as well as subtypes of non-small cell lung cancer. ADC, adenocarcinoma, SCC, squamous cell carcinoma, mADC, mucinous adenocarcinoma. Adjusted p-values for **(C)** and **(E)** were obtained from DESeq2.

To characterize IGFIR-driven lung tumorigenesis at the gene expression level, RNA-Seq was carried out on tumors from SPC-IGFIR transgenic mice and normal lung tissue obtained from non-transgenic animals (Fig 1A). As expected, unsupervised hierarchical clustering of the normalized reads revealed two distinct groups separating the tumor and normal samples (Fig 1B). Differential gene expression analysis identified 4991 genes with altered expression of which a greater proportion were down-regulated (Fig 1C and Fig 1D and S1 Table). Top up-regulated genes included the IGFIR/IR adaptor *Grb14*, the serine-threonine kinase *Stk39* and the EGFR ligands *Ereg* and *Areg* while the most significantly down-regulated genes included those expressed in normal lung cell populations including *Rspo4*, *Myl3* and *Reg3g*. As expected, sequence read alignment against a mouse-human hybrid reference genome confirmed high levels of the human *IGFIR* transgene but not endogenous murine *Igf1r* (Fig 1E).

To identify biological pathways associated with these changes in gene expression, we carried out gene-set enrichment analysis (GSEA)[39]. Using a large collection of gene-sets (~19,000) representing diverse pathways, tumors were enriched for processes related to cell division, cell cycle, DNA repair, translation initiation and lysosomal activity (S1 Fig). Conversely, repressed pathways were largely related to normal lung biology and included chemotaxis as well as muscle and lymphatics development.

### Marker expression of murine IGFIR-driven lung tumors

Tumors from SPC-IGFIR mice feature an adenoma/adenocarcinoma histology [28]. Consistent with this, IGFIR-driven tumors expressed established markers of human lung adenocarcinoma [44] including *Nkx2-1* and *Napsa* while the expression levels of marker genes associated with lung squamous cell carcinoma (*Trp63* and *Krt5*) or mucinous adenocarcinoma (*Muc5ac*, *Muc5b*) were either lower than normal lung tissue or unchanged (Fig 1F). In addition, tumors contained elevated levels of the AT2 cell lineage markers including (*Sftpa1*, *Sftpb*, *Sftpd*) while the club cell marker *Scgb1a1* (also known as *Cc10* or *Ccsp*) was significantly reduced. These findings validate the pathohistology of IGFIR-driven tumors and support AT2 cells as the tumor cell of origin using the SPC-rtTA driver line.

### Comparative gene expression analysis of murine lung tumor models

To explore how the gene expression profile of SPC-IGFIR mice related to other mouse models of lung cancer, we assembled a collection of publically available microarray-based gene expression datasets that contained both tumor and normal lung samples and were representative of diverse lung tumor subtypes (Table 1). As a measure of similarity, we computed the spearman correlation coefficient of log2 fold-changes between genes differentially expressed in each mouse model with the corresponding genes in the SPC-IGFIR model. This analysis revealed a stronger correlation between tumors from SPC-IGFIR mice with models of AD/ADC (spearman’s rho = 0.46–0.82) than the other tumor subtypes (spearman’s rho = -0.0054–0.18). An exception was the moderate correlation of IGFIR-driven tumors and other AD/ADC models with the *Rb/p53* double knockout SCLC model (spearman’s rho = 0.54) which may be reflective of the high expression of cell cycle associated genes.

**Table 1:** Summary of mouse models of lung cancer and gene expression profiles analyzed in this study.

Mouse Model	Gene Promoter	Description	Data Repository	Reference
Kras^LA2^ (K)	spontaneous recombination	Mice carry a latent Kras^G12D^ allele and develop lung AD/ADC	GSE49200	[27]
Urethane (U)	Chemical	Injection of urethane into male A/J mice leads to induction of AD/ADC	GSE2514	[26]
EGFR^mut^ (C/L, C/T, C/L+T)	CCSP-rtTA (rat)	Dox inducible overexpression of mutant versions of EGFR (L858R, T790M or L858R+T790M) leading to AD/ADC	GSE17373	[18]
Myc (M)	SPC (human)	Overexpression of cMyc leading to AD/ADC	GSE54829	[45]
Stat3C (S3)	CCSP-rtTA (rat)	Overexpression of constitutively active Stat3 induces inflammation followed by AD/ADC formation	E-MEXP-1137	[22]
cRaf1 (cR)	SPC (human)	Overexpression of cRaf1 resulting in AD/ADC formation	GSE14277	[21]
Kras^G12D^;Nkx2-1+/- (NK)	CCSP-rtTA (rat) and heterozygous null	Dox inducible overexpression of KrasG12 in a Nkx2-1 heterozygous background leading to mADC	GSE4058	[24]
Pten/Smad4 (PS)	CCSPiCre (endogenous murine)	CKO of Pten and Smad4 in the proximal airways induces ADSQ tumors	GSE47116	[23]
Lkb1/Pten (LP)	Ad-Cre	CKO of Lkb1 and Pten leading to SCC	See Methods	[25]
Rb/p53 (RP)	Ad-Cre	CKO of Rb and p53 resulting in tumors that resemble SCLC	GSE18534	[46]

Ad-cre, Cre recombinase adenovirus; CCSP, club cell secretory protein; CKI, conditional knock-in CKO; conditional knockout; Dox, doxycycline; iCre, improved Cre; rtTA, reverse-tetracycline transactivator; SPC, surfactant-protein C; AD/ADC, adenoma/adenocarcinoma; mAD, mucinous adenocarcinoma; SCC, squamous cell carcinoma; ADSQ, adenosquamous

**Fig 2:**
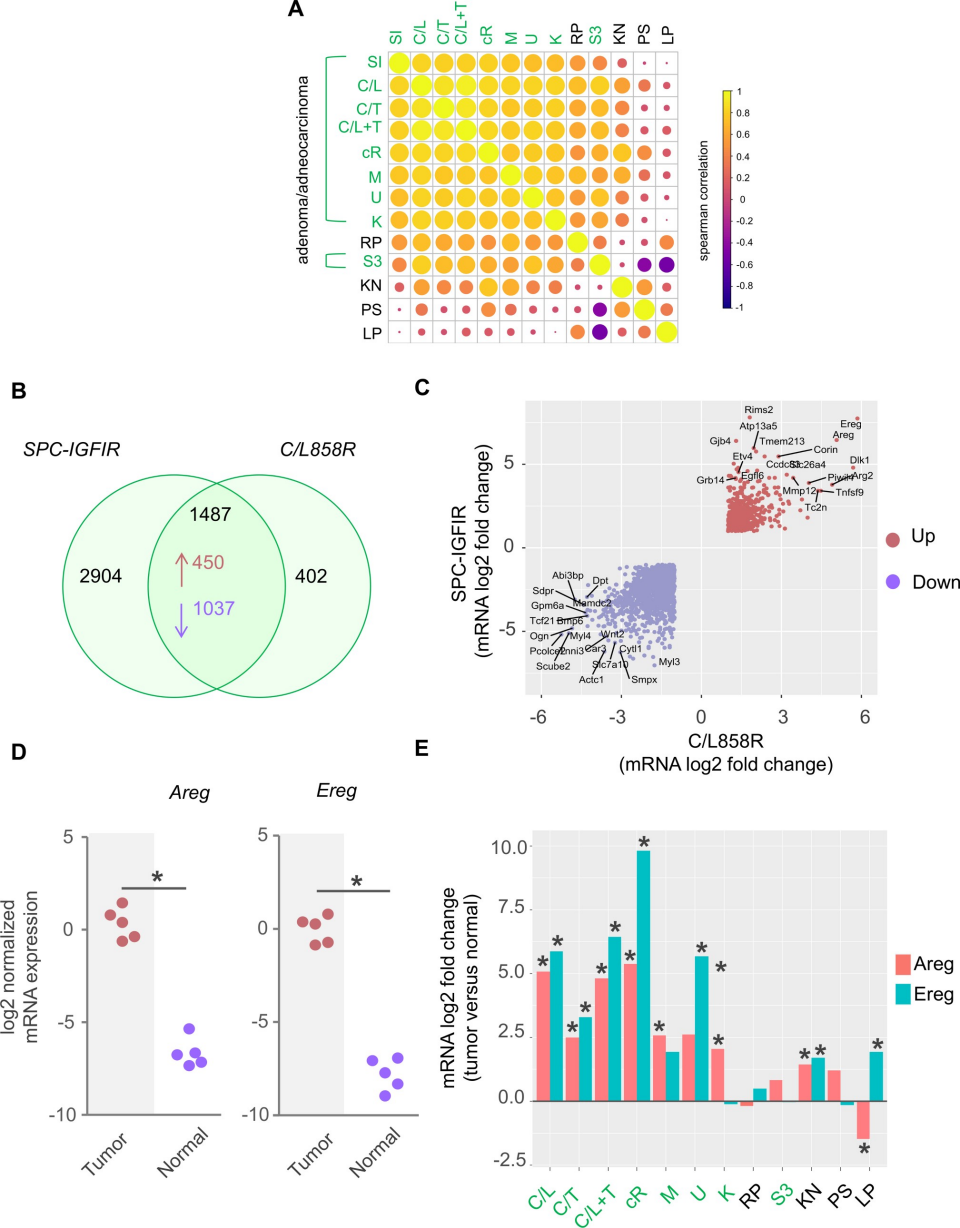
Comparative analysis of mouse models of lung cancer. **(A**) Correlation matrix of log2 fold-changes (tumor vs normal) between the SPC-IGFIR model and mouse models of lung cancer. **(B)** Venn diagram and **(C)** scatterplot of log2 fold changes (tumor vs normal) between the SPC-IGFIR and C/L models. Only genes differentially expressed in the same direction in both the SPC-IGFIR models are plotted. **(D)** Dot plot of *Areg* and *Ereg* mRNA expression levels determined by qPCR (n = 5 for each group). *Ereg*, p <0.0001, *Areg*, p = 0.0004 by 2-tailed t-test. **(E)** Bar plot of *Areg* and *Ereg* log2 fold changes (tumor versus normal) derived from microarray datasets across multiple mouse models of lung cancer. Adenoma/adenocarcinoma (SI, SPC-IGFIR; C/L, CCSP-L858R; C/T, CCSP-T790M; C/L+T, CCSP-L858R+T790M, cR, cRaf1; M, SPC-Myc; U, Urethane; K, Kras^LA2^; S3, CCSP-Stat3C), Small cell lung cancer (RP, Rb/p53 DKO); Mucinous Adenocarcinoma (KN, Kras^G12D^/Nkx2.1^+/-^); Adenosquamous cell carcinoma (PS, Pten/Smad4 DKO); Squamous cell carcinoma (LP, Lkb1/Pten DKO).

Tumors from SPC-IGFIR mice displayed the strongest similarity with the C/L858R model (C/L) [18]; a doxycycline inducible model characterized by overexpression of the EGFR kinase domain mutation L8585R (c.2573T>G) (Fig 2A). Gene overlap analysis identified a total of 1487 differentially expressed genes altered in the same direction between the two models (Fig 2B). Plotting of the log2 fold changes of these shared genes revealed high expression of several genes implicated in human lung cancer including the Notch inhibitor *Dlk1* [38],the matrix metalloprotease *Mmp12* [47] as well as the EGFR ligands *Ereg* and *Areg* which were the two most highly expressed genes in both models (Fig 2C).

The dramatic upregulation of *Ereg* and *Areg* mRNA expression in IGFIR-driven tumors compared to normal lung tissue was confirmed by qPCR (Fig 2D). In addition, significant up-regulation of *Ereg* and/or *Areg* was also identified in a number of other AD/ADC models (Fig 2E). To gain insight into whether tumor cells themselves are the primary source of *Ereg* and *Areg*, we used magnetic cell sorting to isolate IGFIR^+^ tumor and non-epithelial lineage cells (IGFIR^-^/CD31^+^/CD45^+^/Tert119^+^/BP1^+^) containing mixed stromal cell populations (hematopoietic, endothelial, fibroblast) followed by qPCR (S2 Fig). As expected, we observed increased *IGFIR* expression in the tumor cell compartment (S2 Fig). We also found consistent up-regulation of *Areg* and *Ereg* in tumor cells though changes in *Ereg* were not statistically significant. Taken together, these findings indicate that diverse mouse lung tumor subtypes are associated with distinct gene expression profiles and that IGFIR-driven tumors share molecular features with established models of AD/ADC characterized at least in part by a deregulated EGFR pathway.

### Cross-species gene expression comparison with human NSCLC

**Fig 3:**
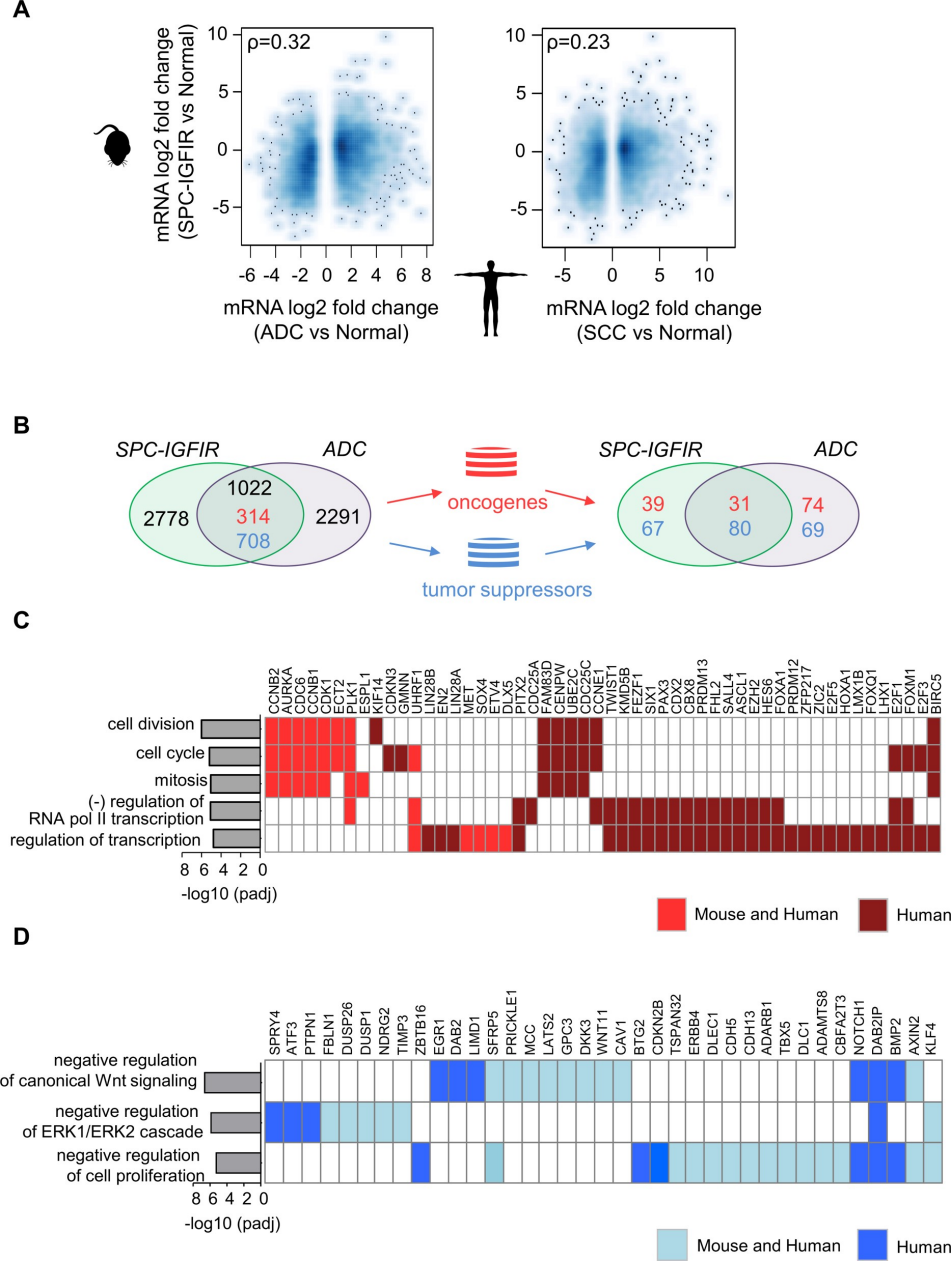
Cross-species analysis of gene expression profiles with human NSCLC. **(A)** Smoothed scatterplots of log2 fold changes of genes differentially expressed in human lung ADC (tumor versus normal) (LUAD, n = 181; N, n = 20) and SCC (LUSC, n = 178; N, n = 59) with orthologous genes in the SPC-IGFIR model. ρ, spearman correlation coefficient (rho). **(B)** Venn diagrams illustrating the identification of genes with coordinated expression across IGFIR-driven and human lung ADC. **(C)** Bar chart with overrepresented gene ontology (GO) biological terms alongside a plot of corresponding genes up-regulated in mouse and/or human lung ADCs present within the ONGene and **(D)** TSG2.0 databases. ADC and LUAD, Adenocarcinoma; SCC and LUSC, squamous cell carcinoma.

Given the similarity of IGF-IR driven tumors with mouse lung ADC we next investigated the extent to which the gene expression profile of IGFIR-driven lung tumors mimic the human disease. RNA-Seq data of human ADC [48], SCC [49] and normal lung tissues were obtained from the TCGA dataset. Differentially expressed genes were identified and the spearman correlation coefficient of log2 fold changes with the orthologous murine genes in the SPC-IGFIR dataset was then computed. In contrast to the comparative mouse model analysis, the similarity of IGFIR-driven tumors with human ADC was more moderate (spearman’s rho = 0.32) but higher than SCC (spearman’s rho = 0.23) (Fig 3A). Gene overlap analysis of the differentially expressed genes in human lung ADC and murine IGFIR-driven tumors identified 1022 genes with the same change in direction representing 32% of genes altered in human ADC (Fig 3C and S2 Table). Gene Ontology (GO) and KEGG pathway analysis with Database for Annotation, Visualization and Integrated Discovery (DAVID)[40] revealed the shared upregulated genes were enriched for terms largely related cell cycle processes while shared down-regulated genes were associated with normal lung function (S3 Fig). Terms and pathways unique to IGFIR-driven tumors consisted mainly of processes related to metabolic processes.

To examine the relevance of these shared mouse and human genes to cancer we mined the ONGene [50] and TSG2.0 [51] databases and identified 31 and 80 genes with purported oncogenic and tumor suppressive properties, respectively (Fig 3B). GO enrichment analysis confirmed the biological roles of these genes in cancer related processes including cell cycle and transcriptional regulation as well as migration and Wnt and MAPK signaling (Fig 3C and Fig 3D). Genes upregulated in both IGFIR-driven tumors and human ADC included those with reported roles in lung cancer such as *AURKA*, *SOX4*, *RET*, *MET* and *ETV4* while those downregulated in both species included *KLF4*, *TIMP3*, *CAV1* and *LATS2*. Of note, a subset of these SPC-IGFIR and human lung ADC shared genes were unique to the SPC-IGFIR model when compared to tumors from C/L858R mice with the transcriptional regulator *DLX5* and the secreted Wnt antagonist *SFRP5* displaying the greatest positive and negative fold changes respectively (S4 Fig).

### Deregulated miRNA-mRNA expression in IGFIR-driven lung tumors

**Fig 4:**
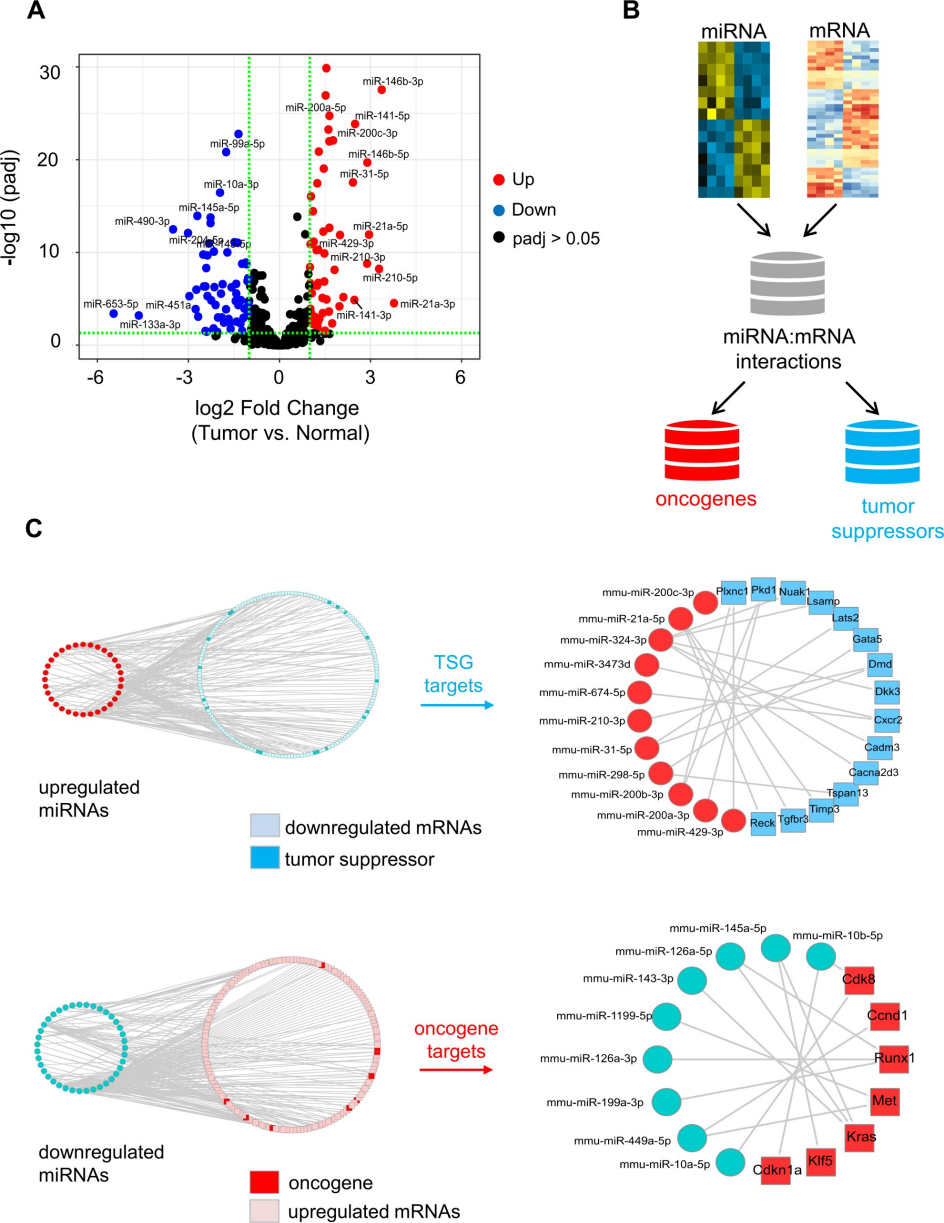
Integrated analysis of miRNA-mRNA expression in IGFIR-driven tumors. **(A)** Volcano plot of log2 fold changes and differential expression p values of miRNAs between tumors from SPC-IGFIR mice and normal lung tissue. **(B)** Schematic of procedure used to identify miRNA:mRNA interaction pairs with oncogenic or tumor suppressive functions. The multiMiR database was used to identify ‘predicted’ and ‘validated’ miRNA targets followed by mining of the ONGene and TSG2.0 databases. **(C)** Regulatory networks of miRNA:mRNA interactions involving ‘validated’ target genes. The networks to the left illustrate all miRNA:mRNA interactions while those to the right highlight miRNA targets reported to have tumor suppressor or oncogenic activity.

MicroRNAs (miRNAs) represent a class of small non-coding RNA molecules (~22nt) that provide an additional layer of gene expression regulation and have been strongly implicated in cancer pathogenesis [52]. Thus, to complement our mRNA analysis, we also carried out small RNA sequencing on tumors from SPC-IGFIR mice and normal lung tissue. Differential expression analysis identified 55 up-regulated and 66 down-regulated miRNAs (fold-change ≥2, FDR<0.05, Fig 4A and S3 Table), many of which have been implicated in cancer [52]. The top up-regulated miRNAs included those with known oncogenic properties (oncomiRs) including *miR-21a-5p/3p*, *miR146b-5p/3p*, *miR-210* and *miR-31-5p* and regulators of the epithelial-state (*miR200b/a/429* and *miR-200c/141*). Down-regulated miRNAs included those with known tumor suppressive activity including *miR-92a-3p*, *miR-99a-5p* and *miR-10a-3p*.

To identify potential miRNA target genes, we integrated the miRNA and mRNA datasets and then accessed multiple databases of predicted and validated miRNA-mRNA interaction using the multiMiR package [53] (S4 Table). Interestingly, pathway analysis revealed these potential miRNA targets were associated with ErbB2 signaling involving ligands (*Ereg*, *Btc*, *Nrg1*, *Nrg2*), receptors (*Egfr*, *Erbb2*, *Erbb3*, *Erbb4*), adaptor proteins (*Shc1*, *Shc4*) and the downstream kinases *Kras*, *Akt3* and *Mapk10* (S5 Fig). Next, we focused on “validated” miRNA-mRNA interactions and further narrowed the list of target genes to those with oncogenic or tumor suppressive functions through mining of the ONGene [50] and TSG2.0 [51] databases (Fig 4B). This led to the identification of 30 miRNA-mRNA interaction pairs (Fig 4C) involving known tumor suppressor targets of *miR-21a-5p* (*Reck*, *Timp3* and *Tgbr3*) and *miR-31-5p* (*Lats2* and *Dmd*) as well as oncogenic targets of *miR-143-3p* (*Kras*), *miR-145-5p* (*Klf5*, *Kras*) and *miR-1195-5p* (*Met)*.

### Comparative miRNA expression

In contrast to the large number of high-throughput gene expression studies carried out on mouse models of lung cancer, few genome-wide miRNA profiles have been described. Although Kras-driven tumors exhibited only a modest correlation with SPC-IGFIR mice at the mRNA level, the availability of miRNA-Seq data led us to explore potential similarities and differences in miRNA expression between these two models. First, we re-analyzed this dataset at the isomiR level as the original study reported total miRNA abundances [38]. Then we compared the log2 fold changes of differentially expressed miRNAs in the Kras^G12D^ model with corresponding miRNAs in SPC-IGFIR mice. Similar to the comparative mRNA expression analysis, a modest correlation of 0.49 was observed with Kras-driven tumors (S6 Fig). Overlap analysis identified a small subset of miRNAs (13 of 115; 10%) differentially expressed in the same direction (log2 fold change >1, FDR <0.05) including *miR-184-3p*, *miR-21-5p*, miR-*31-5p/3p* and *miR-145a-5p* (S6 Fig).

A significant number of miRNAs are located in genomic clusters [54] and in the Kras^G12D^ model, the majority of differentially expressed miRNAs were reported to be located in a cluster at the *Dlk1-Dio3* locus on chr12qF1 [38]. We confirmed these results in our re-analysis of this dataset but in contrast to Kras^G12D^-driven tumors, we found differentially expressed miRNAs in the SPC-IGFIR model were located in clusters along multiple chromosomes with a complete absence of altered miRNAs found on chr12 (S6 Fig). Therefore, these results further demonstrate that despite sharing similar histological features, murine lung ADCs initiated by different oncogenic drivers may be associated with distinct molecular changes including those at the miRNA level.

**Fig 5:**
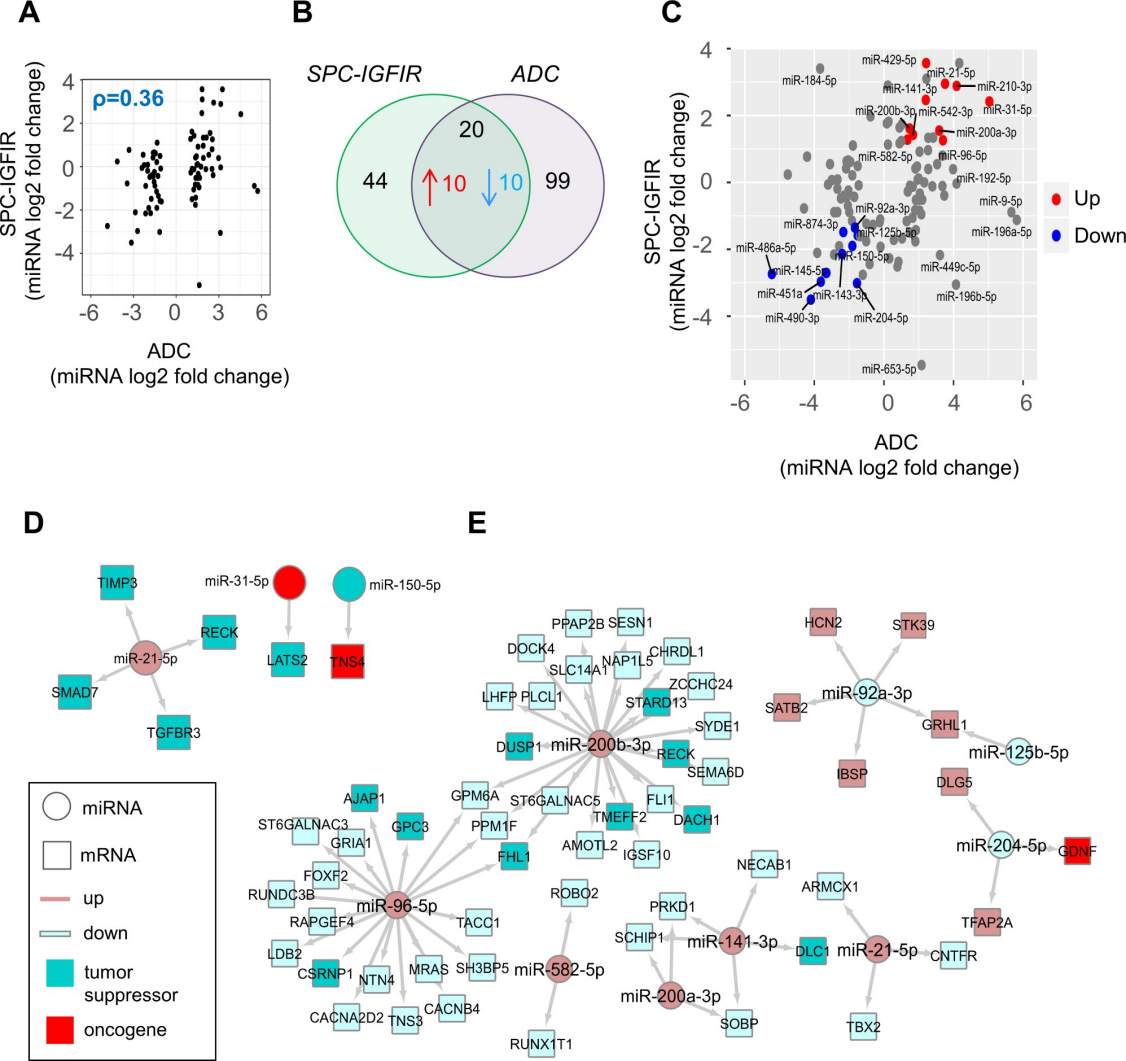
Comparative miRNA analysis. **(A)** Scatter plot of log2 fold changes of differentially expressed miRNAs in human ADC with conserved miRNAs in SPC-IGFIR mice. ρ, spearman’s correlation coefficient (rho). **(B)** Venn diagram illustrating overlap of differentially expressed miRNAs in human ADC and SPC-IGFIR mice. **(C)** Scatter plot of log2 fold changes of miRNAs differentially expressed in SPC-IGFIR mice and human ADC (n = 181 tumor, n = 20 normal). Up and downregulated miRNAs shared across species are highlighted in red and blue respectively. miRNAs highlighted in grey are altered only in one species. **(D)** Network diagram of ‘validated’ and **(E)** ‘predicted’ miRNA:mRNA interactions shared between SPC-IGFIR and human ADC. miRNA targets with reported oncogenic or tumor suppressor functions are highlighted.

Finally, we compared the miRNA expression profile of IGFIR-driven tumors with human lung ADC using miRNA-Seq data of tumor and normal tissue from the TCGA-LUAD dataset [48](S5 Table). Similar to the mRNA comparative analysis, a modest correlation was found (spearman’s rho = 0.36) between differentially expressed miRNAs identified in human ADC and species conserved orthologous miRNAs in the SPC-IGFIR model (Fig 5A). A total of 20 miRNAs with coordinated changes in expression across species were identified including *miR-21-5p*, *miR-31-5p*, *miR-210-3p* and *miR-490-3p* (Fig 5B and Fig 5C). A subset of miRNAs also exhibited discordant expression including upregulation of *miR-184-5p* in IGFIR-driven tumors and increased expression of *miR-9-5p*, *miR-196a-5p miR-653-5p* in human ADC. Integration of the human miRNA-mRNA datasets with multiMiR (S6 Table) followed by mining of the ONGene [50] and TSG2.0 [51] databases identified 6 ‘validated’ interaction pairs common between IGFIR-driven tumors and human ADC involving *miR-21-5p* (*RECK*, *TIMP3*, *TGFBR3*), *miR-31-5p* (*LATS2*) and *miR-150-5p* (*TNS4*) (Fig 5D). A number of shared ‘predicted’ miRNA targets that may also be involved in IGFIR-mediated tumorigenesis and human ADC included the tumor suppressors deleted in liver cancer 1 (*DLC1*), adherens junction associated protein 1 (*AJAP1*) and glypican-3 (*GPC3*) (Fig 5E).

## Discussion

Here we report on the gene expression profile a mouse model of IGFIR-driven lung cancer. The goal of this study was to not only understand the molecular changes associated with IGFIR-driven tumorigenesis but to also assess the relevance of these alterations in the context of other established mouse models of lung cancer and the human disease. In the mouse-to-mouse comparison, we found similarities with models of lung ADCs initiated by different oncogenic drivers, particularly those driven by mutant versions of EGFR. This is perhaps not surprising given that both IGFIR and EGFR are receptor tyrosine kinases (RTKs) that activate common signaling pathways including PI3K/AKT and Ras/MAPK [55]. Despite the similarities in gene expression between these models, there are clear differences in the oncogenic potential of IGFIR and EGFR in the mouse lung as tumor onset occurs much more rapidly in the mutant EGFR models (4–8 weeks versus 8–9 months in SPC-IGFIR mice). This dramatic difference in tumor latency suggests the acquisition of additional collaborating events may play an important role in the initiation of IGFIR-driven lung tumors and that the SPC-IGFIR model may be of value in identifying such alterations.

Upregulation of the EGFR ligands *Ereg* and/or *Areg* was a common feature between IGFIR-driven tumors and mouse models of lung ADC. Elevated levels of these ligands has been previously noted in a subset of these mouse models [18,21,56]. Interestingly, loss of *Ereg* reduced tumor burden in a carcinogen induced mouse model of lung cancer [56] but was not required for intestinal tumorigenesis in APC^min^ mice [57] suggesting a tumor-type specific role for this ligand. In human lung cancer, EREG and AREG mRNA or protein levels are elevated in a subset of NSCLC cell lines [58,59] as well as NSCLC specimens and predict poor survival [59,60]. Expression of *EREG* mRNA has also been found to be higher in adenocarcinomas versus squamous cell carcinomas [59]. Although classically thought of as epithelial specific molecules that regulate EGFR signaling via an autocrine loop, production of EREG and AREG has been detected in stromal cells including normal and cancer associated fibroblasts [61–63] and various immune cell populations [64,65]. Using magnetic cell sorting and qPCR we also observed expression of *Ereg* and *Areg* in the tumor and non-epithelial cell compartment though levels were significantly higher (*Areg*) or trended towards being higher (*Ereg*) in the tumor cell population. A recent computational analysis of tumor-stroma interactions in murine Kras-driven lung tumors identified an Ereg-Egfr autocrine signaling axis in tumor cells and an Areg-Egfr pathway of monocyte-tumor cell crosstalk [66]. Given that these ligands may represent alternative targets for therapeutic inhibition of EGFR signaling in lung cancer [66] and the growing importance of immune effectors in lung cancer pathogenesis, the specific cell types that express *Ereg* and *Areg* and their functional role in tumorigenesis are areas of interest in the future use of the SPC-IGFIR model.

In a cross-species analysis, we observed a more modest similarity in gene expression (32% of differentially expressed genes) between IGFIR-driven tumors and human lung ADC compared to those computed for mouse models of lung ADC. This finding was not unexpected given the significant molecular heterogeneity evident in the majority of human solid cancers including lung ADC [3,48,67]. These results are also consistent with a cross-species study of urethane-induced tumors from Stearman and colleagues in which an overlap of only 409 genes (representing approximately 15% of altered genes in human lung ADC) was observed [26]. Nevertheless, the utility of this model was validated as conserved changes in genes relating to glycolysis, cell cycle and the eicosanoid pathway were discovered. In addition, cross-species analysis of the Kras^LA2^ model revealed approximately 12% of differentially expressed genes were shared with human lung ADC and when combined with GSEA led to the identification of a gene signature of human *KRAS*-mutant tumors which could not be identified from the analysis of human *KRAS*-mutant alone [27]. Thus, while mouse lung cancer models may not recapitulate all of the gene expression changes observed in human tumors, a subset of conserved alterations are often present that can provide important insight into the disease.

In this study, we focused on the identification of species conserved changes in cancer-related genes resulting in the identification of a number of alterations with oncogenic and tumor suppressive properties that were altered across species. Of interest is the shared upregulation of *DLX5* (Distal-less Homeobox 5), which did not appear to be altered in the C/L858R EGFR-driven model. DLX5 is one of six DLX family members expressed during embryonic development [68] but has also been implicated in a number of cancers [69–72]. In NSCLC, DLX5 has been reported to be overexpressed at the mRNA and protein level, and correlated with tumor size and poor prognosis [72]. At the mechanistic level, the pro-tumorigenic functions of DLX5 have been shown to involve positive regulation of the *MYC* promoter in human lung cancer cells [70] as well as the *IRS2* promoter, an IGFIR adapter protein, in ovarian cancer cell lines [69]. Whether DLX5 or its downstream targets are required for lung tumor initiation and maintenance in vivo is unknown but our data suggests the SPC-IG1R model may be useful to test this hypothesis.

Deregulated miRNA expression regulates a variety of biological processes related to cancer pathogenesis [52]. Integration of miRNA-mRNA data from IGFIR-driven tumors revealed regulation of pathways including ErbB signaling. In human lung ADC, miRNAs have also been shown to regulate the EGFR pathway and may have potential as biomarkers for predicting anti-EGFR therapeutic response[73]. In a cross-species analysis, we identified miRNAs commonly deregulated in lung ADC including the well-studied oncomiR *miR-21-5p*. Elevated *miR-21* is a defining feature of multiple cancers including lung ADC and is observed more frequently in patients with EGFR mutant disease [73]. The pro-tumorigenic properties of *miR-21* have been linked with suppression of negative regulators of Ras signaling including *Spry1* and *Spry2* [74]. Here, we identified the validated *miR-21* targets *Reck*, *Timp3* and *Tgfbr3* were deregulated in IGFIR-driven tumors which was also conserved in human lung ADC. While *miR-21* and *miR-31* have been shown to play a direct role in regulating Kras-driven lung tumorigenesis in vivo[74,75], whether they play a similar role in lung cancer driven by other oncogenic drivers, including IGFIR, remains unknown. Given that few miRNAs have been directly validated in lung cancer initiation and progression in vivo, the results of this study provide a framework to address the functional role of less well studied miRNAs in lung cancer.

A common theme of this study was the identification of a potential role for the ErbB pathway in IGFIR-driven tumors. EGFR and IGFIR are often co-expressed in patient samples [76] and up-regulation of IGFIR has been shown to mediate EGFR TKI resistance [77]. Pre-clinical studies also demonstrated greater anti-tumor activity with dual targeting of EGFR and IGFIR though clinical translation was not successful [78]. As the clinical failure of anti-IGFIR therapeutics is thought to be due in part to poor patient selection [79], the potential efficacy of combined targeting both pathways remains uncertain. Thus, the SPC-IGFIR model may provide a valuable platform to dissect co-operation between EGFR and IGFIR and also serve as a pre-clinical model to test therapeutic blockade of these signaling pathways.

In summary, we have presented a detailed overview and inter- and cross-species comparative mRNA and miRNA gene expression analysis of IGFIR-driven lung cancer. The comprehensive molecular profiling presented in this study will facilitate future investigations into multiple aspects of tumor initiation and progression and hopefully provide insight towards the identification of rationale therapeutic targets for lung cancer.

## Supporting information

S1 Fig Pathway analysis of IGFIR-driven tumors reveals similarities with mouse and human non-small cell lung cancer. **(A)** GSEA of SPC-IGFIR tumors versus normal lung tissue with enrichment map for visualization. Nodes (circles) in red represent pathways enriched in IGFIR tumors while nodes in blue represent pathways enriched in normal lung tissue. Node size reflects the number of genes in each pathway.
(TIF)

S2 Fig Areg and Ereg mRNA expression in tumor cell populations in SPC-IGFIR mice. **(A)** Diagram illustrating magnetic cell sorting strategy to isolate IGFIR^+^ tumor cells and lineage-negative (Lin^-^, mixed stromal) populations from SPC-IGIR mice. **(B)** Dot plots of *Igf1r*, *Ereg* and *Areg* mRNA expression detected by qPCR in magnetically sorted tumor (IGFIR^+^) and non-epithelial lineage cells from SPC-IGFIR mice (bottom, n = 3 for each group). *Igf1r*, p = 0.034; *Ereg*, p = 0.0928; *Areg*, p = 0.005 by 2-tailed paired t-test.
(TIF)

S3 Fig Comparative functional enrichment analysis of SPC-IGF1R and human ADC. The overlap of differentially expressed genes in human ADC and orthologous genes in the SPC-IGFIR model was calculated followed by gene ontology (GO) and KEGG enrichment analysis using DAVID. Significant terms (padj < 0.05) enriched in genes with coordinated expression (shared) as well as those differentially expressed and unique to each species are plotted.
(TIF)

S4 Fig Comparative analysis of oncogene and tumor suppressor gene expression in IGF1R and EGFR-driven mouse lung tumors. Heatmaps of log2 fold changes of oncogenes **(A)** and tumor suppressor genes **(B)** in tumors from SPC-IGFIR and C/L858R mice compared to normal lung tissue. All genes depicted were first identified as having significant coordinated mRNA expression in SPC-IGFIR mice and human lung ADC from the TCGA dataset. Genes differentially expressed only in SPC-IGFIR mice or in both mouse models are indicated and sorted by log2 fold change.
(TIF)

S5 Fig miRNA regulation of the ErbB pathway in IGF1R-driven tumors. **(A)** Differentially expressed miRNAs and mRNAs were integrated using the multiMiR database followed by functional enrichment of target miRNAs with DAVID. Enriched biological processes of predicted and validated miRNA target genes are shown. **(B)** Network visualization of miRNA-mRNA interactions involved in ErbB signaling.
(TIF)

S6 Fig Comparative analysis of miRNA expression between Kras and IGF1R-driven lung adenocarcinoma. **(A)** Scatter plot of log2 fold changes of miRNAs differentially expressed in Kras^G12D^-driven lung cancer with corresponding miRNAs in IGFIR-driven tumors. **(B)** Venn diagram and **(C)** scatter plot of differentially expressed miRNAs in tumor and normal tissue from SPC-IGFIR and Kras^G12D^ mouse lung tumor models. miRNAs altered in the same direction in both models are highlighted in red (upregulated) and blue (downregulated). **(D)** Bar plot depicting chromosomal location of altered miRNAs in tumors from SPC-IGFIR (top) and Kras^G12D^ (bottom) mice.
(TIF)

S1 Table Differentially expressed genes between lung tumors from SPC-IGFIR mice and non-transgenic normal lung tissue. (XLSX)

S2 Table Differentially expressed genes shared across species between tumor and normal lung tissue from SPC-IGFIR mice and human lung adenocarcinoma. (XLSX)

S3 Table Differentially expressed miRNAs between lung tumors from SPC-IGFIR mice and non-transgenic normal lung tissue. (XLSX)

S4 Table Target genes of altered miRNAs also exhibiting differential expression between lung tumors from SPC-IGFIR mice and non-transgenic normal lung tissue. (XLSX)

S5 Table Differentially expressed miRNAs between human lung adenocarcinoma and adjacent normal tissue from the TCGA dataset. (XLSX)

S6 Table Target genes of altered miRNAs also exhibiting differential expression between human lung adenocarcinoma and normal lung tissue from the TCGA dataset. (XLSX)
