# Supplementary figures and images for: Comparative mRNA and miRNA transcriptome analysis of a mouse model of IGFIR-driven lung cancer
Source: PLoS One. 2018 Nov 9;13(11):e0206948. doi: 10.1371/journal.pone.0206948 (PMC6226179; doi:10.1371/journal.pone.0206948)

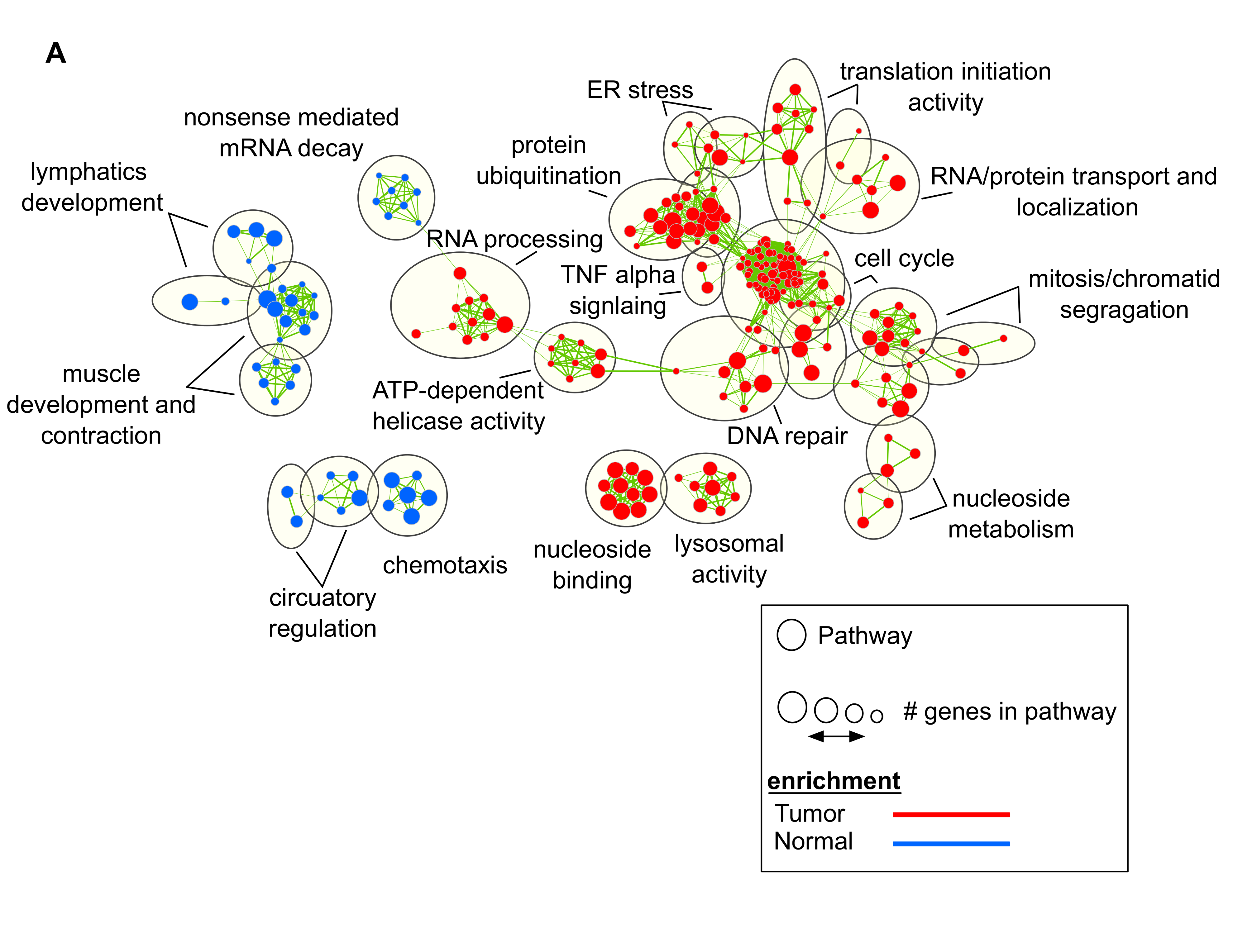

Supplement: S1 Fig — (A) GSEA of SPC-IGFIR tumors versus normal lung tissue with enrichment map for visualization. Nodes (circles) in red represent pathways enriched in IGFIR tumors while nodes in blue represent pathways enriched in normal lung tissue. Node size reflects the number of genes in each pathway. (TIF) [file pone.0206948.s001.tif]

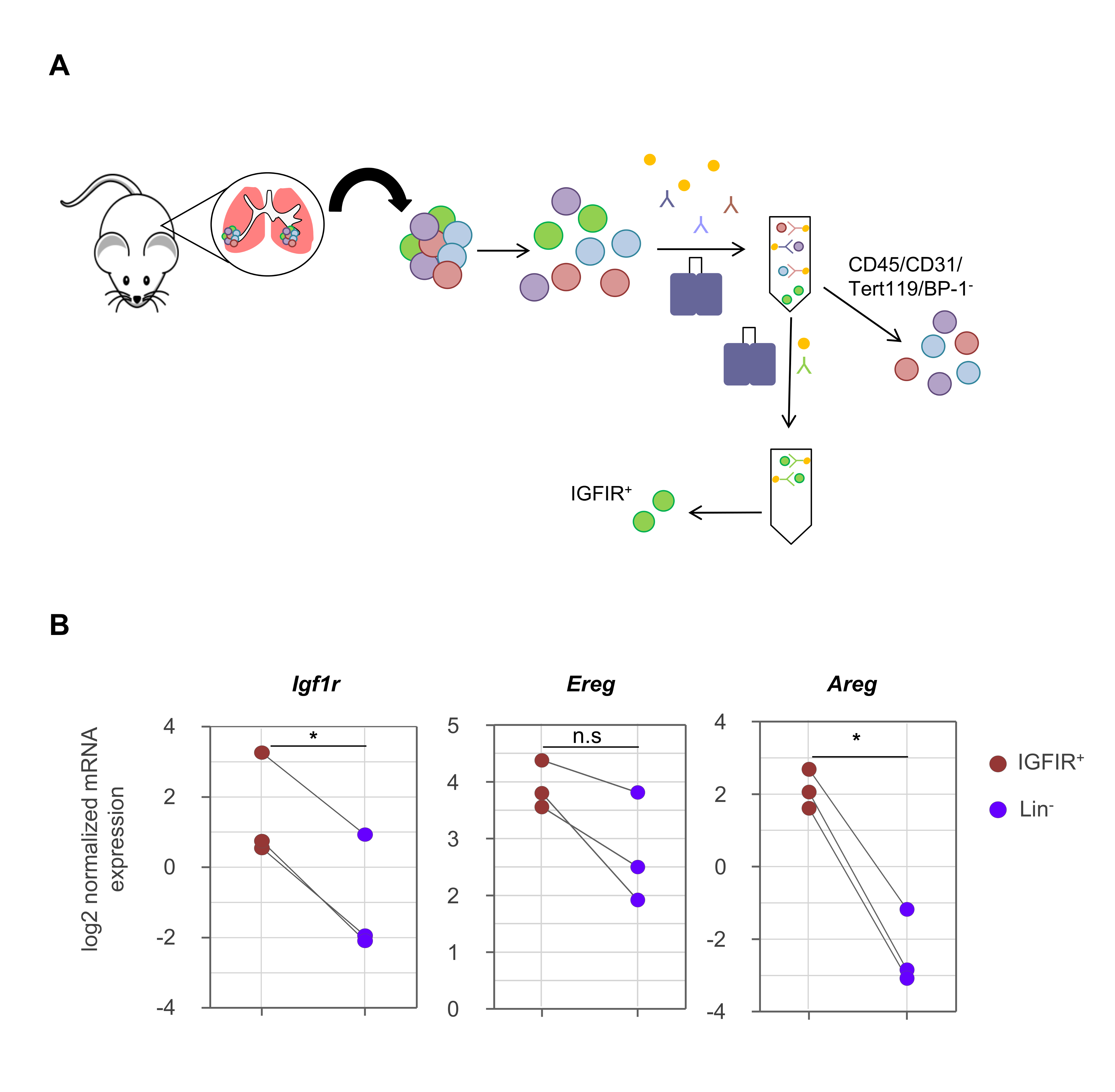

Supplement: S2 Fig — (A) Diagram illustrating magnetic cell sorting strategy to isolate IGFIR+ tumor cells and lineage-negative (Lin-, mixed stromal) populations from SPC-IGIR mice. (B) Dot plots of Igf1r, Ereg and Areg mRNA expression detected by qPCR in magnetically sorted tumor (IGFIR+) and non-epithelial lineage cells from SPC-IGFIR mice (bottom, n = 3 for each group). Igf1r, p = 0.034; Ereg, p = 0.0928; Areg, p = 0.005 by 2-tailed paired t-test. (TIF) [file pone.0206948.s002.tif]

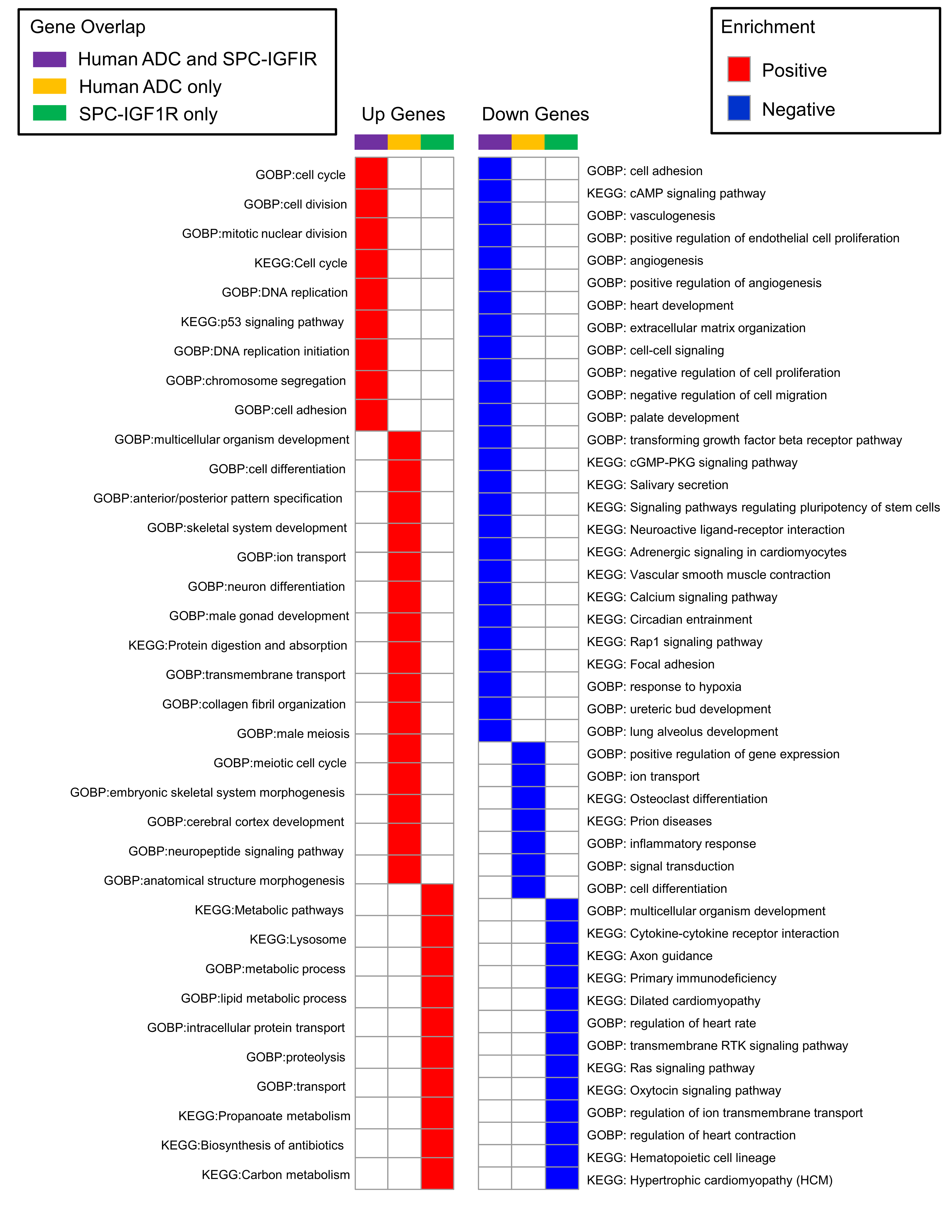

Supplement: S3 Fig — The overlap of differentially expressed genes in human ADC and orthologous genes in the SPC-IGFIR model was calculated followed by gene ontology (GO) and KEGG enrichment analysis using DAVID. Significant terms (padj < 0.05) enriched in genes with coordinated expression (shared) as well as those differentially expressed and unique to each species are plotted. (TIF) [file pone.0206948.s003.tif]

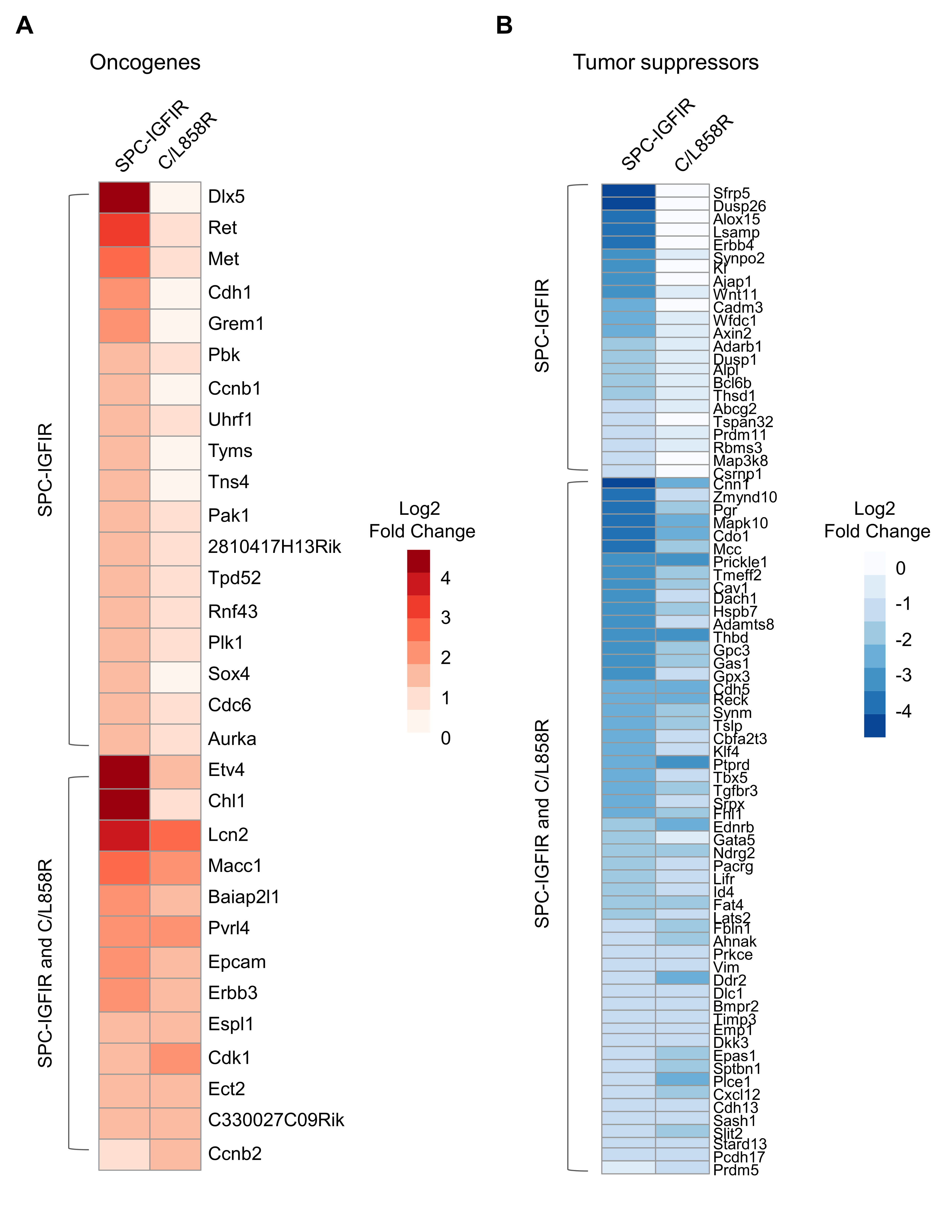

Supplement: S4 Fig — Heatmaps of log2 fold changes of oncogenes (A) and tumor suppressor genes (B) in tumors from SPC-IGFIR and C/L858R mice compared to normal lung tissue. All genes depicted were first identified as having significant coordinated mRNA expression in SPC-IGFIR mice and human lung ADC from the TCGA dataset. Genes differentially expressed only in SPC-IGFIR mice or in both mouse models are indicated and sorted by log2 fold change. (TIF) [file pone.0206948.s004.tif]

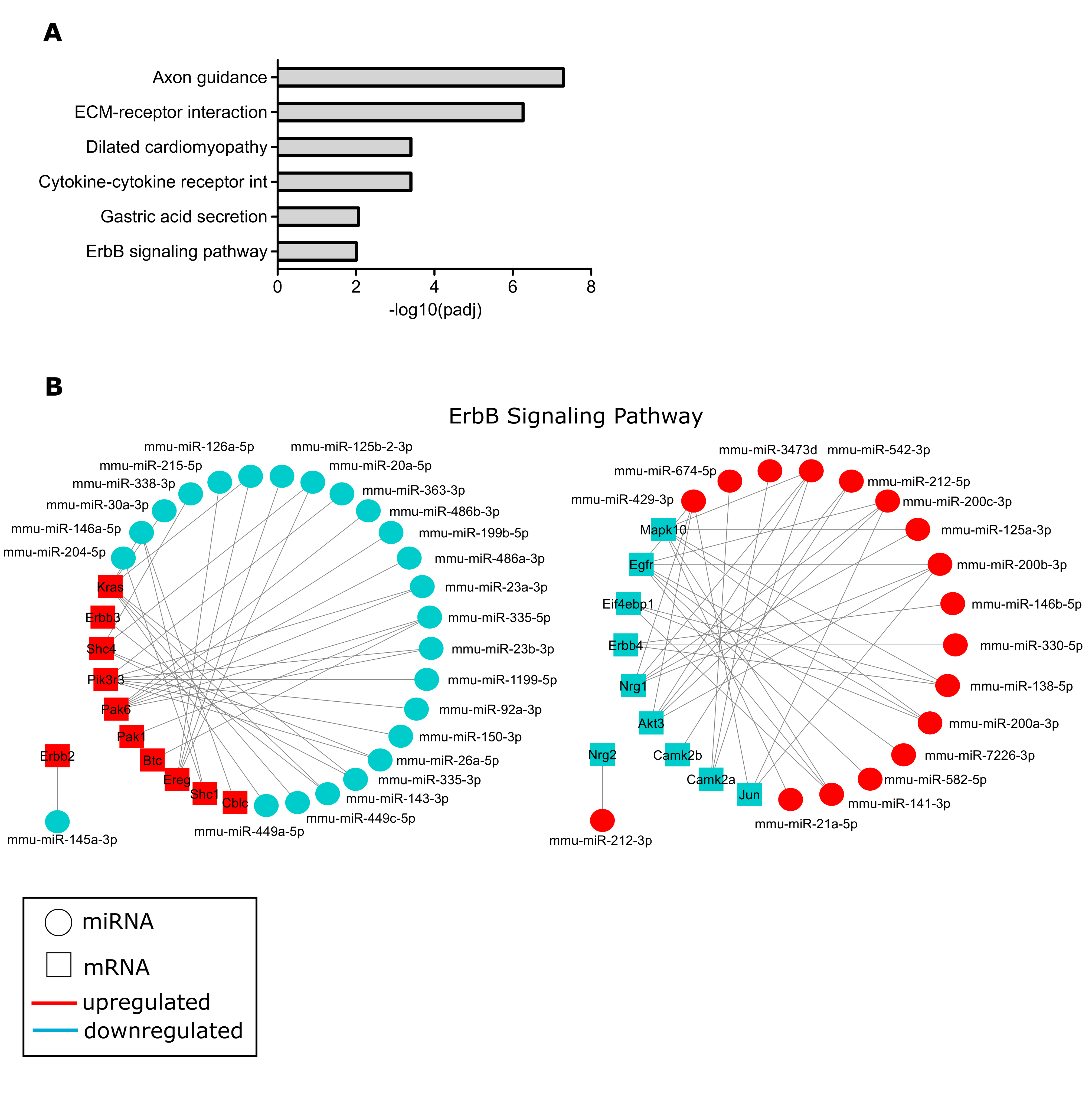

Supplement: S5 Fig — (A) Differentially expressed miRNAs and mRNAs were integrated using the multiMiR database followed by functional enrichment of target miRNAs with DAVID. Enriched biological processes of predicted and validated miRNA target genes are shown. (B) Network visualization of miRNA-mRNA interactions involved in ErbB signaling. (TIF) [file pone.0206948.s005.tif]

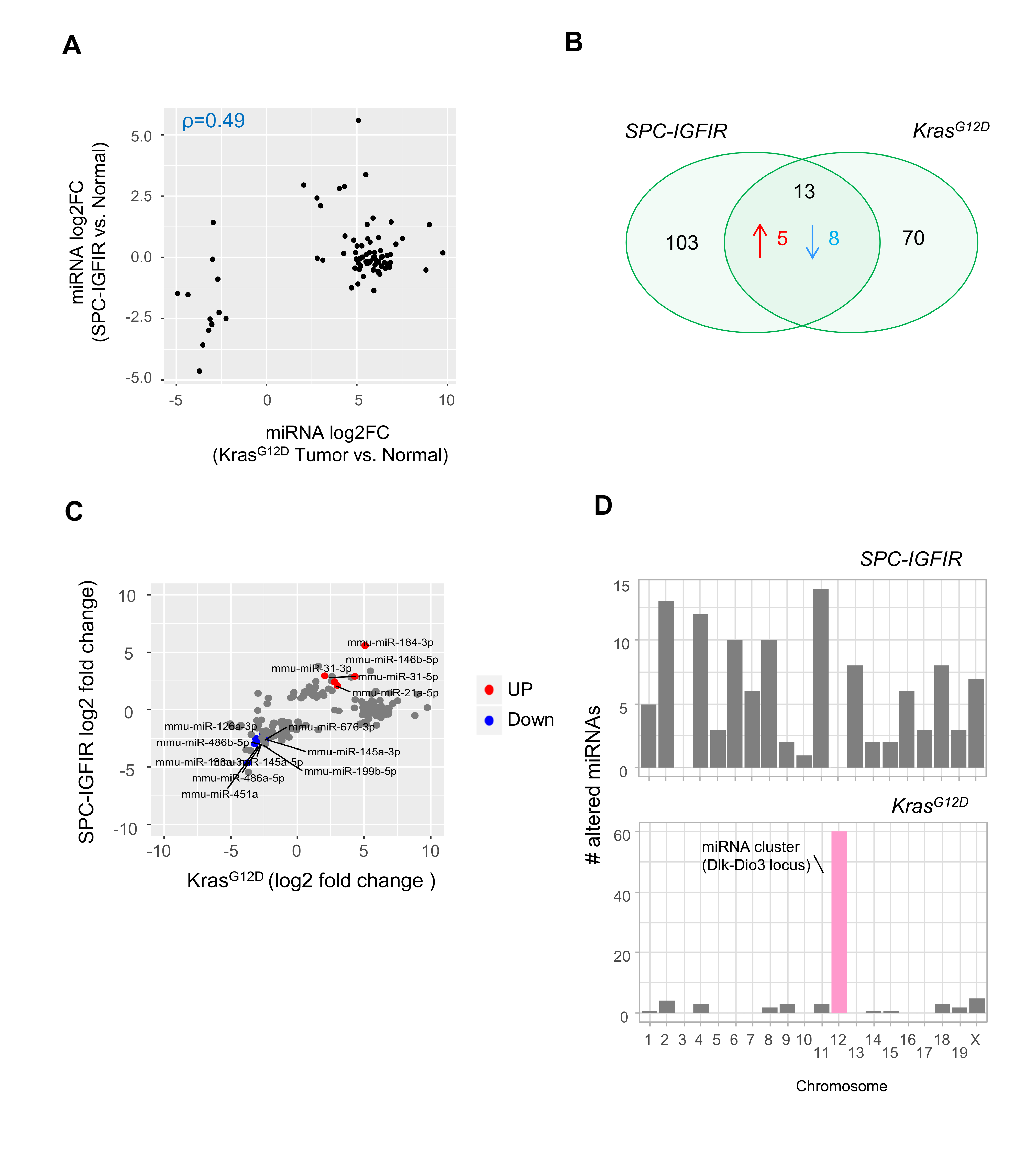

Supplement: S6 Fig — (A) Scatter plot of log2 fold changes of miRNAs differentially expressed in KrasG12D-driven lung cancer with corresponding miRNAs in IGFIR-driven tumors. (B) Venn diagram and (C) scatter plot of differentially expressed miRNAs in tumor and normal tissue from SPC-IGFIR and KrasG12D mouse lung tumor models. miRNAs altered in the same direction in both models are highlighted in red (upregulated) and blue (downregulated). (D) Bar plot depicting chromosomal location of altered miRNAs in tumors from SPC-IGFIR (top) and KrasG12D (bottom) mice. (TIF) [file pone.0206948.s006.tif]
